# Role of Litter Turnover in Soil Quality in Tropical Degraded Lands of Colombia

**DOI:** 10.1155/2014/693981

**Published:** 2014-02-13

**Authors:** Juan D. León, Nelson W. Osorio

**Affiliations:** Universidad Nacional de Colombia, Calle 59 A No. 63-20, Oficina 14-330, 050034 Medellín, Colombia

## Abstract

Land degradation is the result of soil mismanagement that reduces soil productivity and environmental services. An alternative to improve degraded soils through reactivation of biogeochemical nutrient cycles (via litter production and decomposition) is the establishment of active restoration models using new forestry plantations, agroforestry, and silvopastoral systems. On the other hand, passive models of restoration consist of promoting natural successional processes with native plants. The objective in this review is to discuss the role of litter production and decomposition as a key strategy to reactivate biogeochemical nutrient cycles and thus improve soil quality in degraded land of the tropics. For this purpose the results of different projects of land restoration in Colombia are presented based on the dynamics of litter production, nutrient content, and decomposition. The results indicate that in only 6–13 years it is possible to detect soil properties improvements due to litter fall and decomposition. Despite that, low soil nutrient availability, particularly of N and P, seems to be major constraint to reclamation of these fragile ecosystems.

## 1. Introduction

Soil degradation is the result of soil mismanagement reducing soil productivity and environmental services [1, 2]. The most common factors involved in land degradation are soil erosion, deforestation, overgrazing, overtillage, and surface mining [2–4]. According to the World Economic Forum, 60% of the earth's ecosystem services have been degraded in the past 60 years. In the tropics soil degradation affects 500 million ha [5], threatening ecosystem services and food security for people in developing countries [6]. Also, the lack of proper practices (monocultures, inadequate fertilization, lack of soil conservation practices, and reduced tillage) contributes to degradation of soil [3, 7–9]. Soil degradation implies a loss of soil organic matter, structure, porosity, water infiltration and permeability, and nutrient availability, among other considerations [10, 11]. In most of the cultivated land of the tropics the horizon O (organic materials) has disappeared and the horizon A has been severely diminished, organic amendments are rarely used and little is done to reuse crop residues. This impact is particularly severe in the land subjected to surface mining because of the loss of all soil horizons (O, A, B, and C) to expose under layer materials (rocks or sediments) [11]. In both scenarios the biogeochemical cycles of nutrients have been broken leading thus to more soil degradation and increasing the dependence on inorganic fertilizers [12].

An alternative to improve soil quality of degraded lands is the establishment of new forestry plantations, agroforestry, and silvopastoral systems [13], which improve ecosystem services such as: litter supply, nutrient cycling, water infiltration, control of erosion, and increasing of biodiversity [14–19]. This occurs due to (i) the soil exploration by abundant root system, (ii) the protection of the soil surface against erosion, and (iii) the reactivation of nutrient cycling via litter production and decomposition [20–23]. Unfortunately, little is known about the impact of these alternatives on tropical soil parameters. Our hypothesis is that in relatively short periods of time soil quality parameters (e.g., soil pH, soil organic matter content, and plant nutrient availability) may be enhanced in degraded lands by the establishment of forestry plantations or agroforestry systems. Our objective was to review the role of litter turnover, from case studies in diverse ecological life zones of Colombia, as a key strategy to reactivate biogeochemical nutrient cycles and thus improve soil quality in degraded lands of the tropics.

## 2. Experimental Sites

We selected four separate experimental sites in Colombia (Piedras Blancas, Santa Fe de Antioquia, Cáceres, and Cereté) in which land was severely degraded by diverse factors and exhibited contrasting climates and altitude ranging from dry or wet lowlands (18–560 m of altitude) to moist highlands (*∼*2500 m) (Table 1). In each site, forest plantations or silvopastoral systems were established as a way of productive rehabilitation of these environments and were separately studied in diverse projects [5, 7, 13, 24, 25]. Geographic location, weather conditions, land uses, and soil types are provided in Table 1. In the next sections we will discuss some principles of land rehabilitation, litter production and decomposition, nutrient recycling, and changes in soil parameters over time. The results obtained from these experimental sites will be used to illustrate the dynamics of land rehabilitation in the tropics.

## 3. Litter Production and Decomposition

Fine litter production and decomposition are two important processes that provide the main input to form soil organic matter and regulate nutrient cycling in forest ecosystems [26]. The rates at which both processes occur determine the thickness of the litter layer on the forest soil [17]. Nutrient cycling in forestry systems is achieved when the fine litter is decomposed by soil biota, which determines forest primary productivity [27]. Thus, the role of litter in plant nutrition is determined by its turnover time [28]. In fact, in tropical leached soils, the standing litter satisfies most of nutritional need of trees, as dense root systems are developed inside of it [29–31].

In degraded lands by mining activities, the loss of litter and plant coverage on the soil surface disrupts biogeochemical cycles of nutrients [10]. Land reclamation of these soils may be achieved by establishing forestry species, which must be chosen based on their ability to adapt to extreme and restrictive soil conditions [11].

## 4. Models of Land Restoration

Passive and active restoration models have been proposed to restore the functioning of ecological processes [9] in degraded lands. Passive restoration models are based on natural succession processes with minimal human intervention, while active restoration models include planting trees at high density and their respective management [32]. These restoration models may contribute to the amelioration of degraded soils through fine litter production and decomposition as sources of organic matter and nutrients [12].

Although passive restoration models are simple, inexpensive, and based on natural regeneration, these processes are not always successful [33, 34]. Alternatively, active restoration models accelerate the restoration of ecosystem functioning through the activation of soil biogeochemical cycling of plant nutrients and carbon sequestration [32]. Several studies have demonstrated that forestry plantations (active model) play an important role in the improvement of soil quality [35–39].

In general, an active model should be considered when the rate of degradation of the area of interest is high, because the planted species can be established quickly and create better conditions for a more diverse biological community. When the state and rate of degradation are not severe, the most appropriate model might be the passive restoration, allowing a natural recovery of the ecosystem, which had advantages from ecological and economic perspectives [9, 40, 41].

An example of a successful active model in the humid tropics is the establishment of plantations of *Acacia* genus (*A. albida, A. Senegal*, and* A. mangium*) in land reclamation [42]. *A. mangium* grows quickly and has a high capacity to adapt to nutrient-poor acidic soils, due to its capacity to establish symbiotic associations with N_2_ fixing bacteria [43–45] and mycorrhizal fungi [46, 47]. In extremely nutrient-poor soil *A*. *mangium* has exhibited an outstanding growth rate higher than other plant species such as *Eucalyptus *sp. and *Gmelina arborea* [11].

In tropical dry regions some other forest species have been planted for land reclamation. This is the case of *Azadirachta indica *A. Juss (Neem), a plant species employed broadly single or in mixed plantations; its high-quality litter and rapid decomposition promote its use for improving soil quality in rocky and sandy lands that are prone to desertification [48], in soils degraded by surface mining [49, 50] and in soils affected by salinity [51].

## 5. Biogeochemical Nutrient Cycling Considerations Applied to Land Restoration

Regardless of the restoration models used several aspects related to nutrient cycling must be seriously considered. First, the major source of soil organic matter in terrestrial ecosystems is the fine litter production. The diverse plant tissues that compose fine litter (leaves, small branches, flowers, fruits, and seeds) are accumulated on the soil surface and must be decomposed to release nutrients. In many tropical soils, nutrients released from the litter are the most relevant source of plant nutrients [31] and humus formation [52, 53]. In this way, from a functional perspective, the standing litter on the soil surface is very important in the regulation of several processes [54] that include soil protection against erosion [55]. The rate of litter decay controls soil organic matter formation and nutrient input.

The leaves constitute the most abundant and easily decomposable fraction of the fine litterfall (~70%) [56]; consequently, the rate at which leaf litter is produced and decomposed represents a significant aspect in restoration programs of degraded soils. For this reason, plant species with abundant leaf litter production of rapid decomposition must be considered in such programs. Therefore, a low residence time of leaf litter seems to be a key factor in the reactivation of biogeochemical nutrient cycles in degraded soils [57].

The rate of leaf litter production is lower in tropical highland forests than in tropical lowland forests. In the first case, the values fluctuates 9-10 t ha^−1^ yr^−1^ [61–64], while in lowlands the values are over 13 t ha^−1^ yr^−1^ [65, 66]. This input of organic matter and nutrients favors the activity of soil micro- and mesobiota, which controls not only ecosystem functioning and productivity via nutrient cycling but also the ecosystem resilience against disturbance.

Litter accumulation on soil surface results in a balance between litter production and decomposition. In highland, total forest litter accumulation ranges between 10 and 17 t ha^−1^ [28, 52, 67]. The leaves usually were around 2–7 t ha^−1^ [52, 64, 67, 68].

## 6. Organic Matter Return Based on Litter Production and Accumulation

In early works Jenny et al. [69] proposed to measure the rate of litter decomposition based on litter fall values and its accumulation. Thus, in tropical rain forest the litterfall and decomposition is continuous and practically constant. In short periods of time (*dt*) it is accepted that there is an equilibrium between production and decomposition (2):

(1) $$\begin{array}{c} A\;dt=k_{j}\left(F+A\right)dt, \end{array}$$

or

(2) $$\begin{array}{c} A=k_{j}\left(F+A\right), \end{array}$$

where *A*: annual rate of litterfall (Mg ha^−1^ yr^−1^), *dt*: time difference, *k*_*j*_: constant that represents the litter fraction that decays, and *F*: litter amount accumulated before the measuring of litterfall (Mg ha^−1^ yr^−1^). Thus, at equilibrium, the litter loss (decomposition) is compensated by the litter additions (litter production):

(3) $$\begin{array}{c} k_{j}=\frac{A}{\left(F+A\right)}. \end{array}$$

The mean residence time (MRT_*j*_) of the litter and the nutrients in it can be estimated from the inverse of *k*_*j*_:

(4) $$\begin{array}{c} {\text{MRT}}_{j}=\frac{1}{k_{j}}=\frac{\left(F+A\right)}{A}. \end{array}$$

Consequently, the leaf litter real return (LLRR) to soil can be estimated as the product of annual leaf litter potential return (*A*) and *k*_*j*_:

(5) $$\begin{array}{c} \text{LLRR}=Ak_{j}. \end{array}$$

It should be clear that in this estimation leaf litter (*A*) represents the most important source for turnover of organic matter in the ecosystem; other organic materials in the litter fall such as flowers, fruits, and woody debris and above ground litter accumulated previously (*F*) are ignored. On the other hand, other processes such as herbivory, volatilization, leaching, runoff, and microbial immobilization represent losses of organic matter that are not considered. Therefore, this approach likely underestimates/overestimates the total turnover that occurs in the ecosystem.

According to Nye [70], once the litter accumulates up to reach an equilibrium state, the rate of litter addition in *dt* will be equal to the rate of litter loss in the same time period (6). From this equation the index *k*_*L*_ is found (7):

(6) $$\begin{array}{c} A\;dt=k_{L}F\;dt, \end{array}$$

(7) $$\begin{array}{c} k_{L}=\frac{A}{F}. \end{array}$$

Values of *k*_*L*_ > 1 indicate a return of the litter layer below one year [68]. The mean residence time (MRT_*L*_) of the litter and the nutrients in it can be estimated from the inverse of *k*_*L*_:

(8) $$\begin{array}{c} {\text{MRT}}_{L}=\frac{1}{k_{L}}=\frac{F}{A}. \end{array}$$

The litter accumulated in a forestry ecosystem usually has lesser proportion of leaves than the litter that felt; this suggests a fragmentation of the original material to form *unidentified material* due to a rapid decomposition of labile organic materials and nutrient release into the soil. León et al. [52] compared biogeochemical cycles of nutrients in an oak (*Quercus humboldtii*) forest and a *Pinus patula* plantation established in a degraded soil by overgrazing in the Andean mountains of Antioquia, Colombia. The values of the decomposition constant for leaf litter, *k*_*j*_, ranged between 0.58 (oak) and 0.42 (pine), respectively (Table 2). These values were lower than those reported for tropical lowland forests (1.4–2.0 yr^−1^) [71, 72] and for tropical highland forests (2.0 yr^−1^) [73]. The MRT_*j*_ of the leaf litter was 1.72 yr for the oak forest and 2.36 yr for the pine plantation. The differences in the rate of decomposition may be explained in terms of the C/N ratio (oak leaves = 39; pine leaves = 51). Torreta and Takeda [74] considered that critical values of C/N ratio for litter ranged between 30 and 40. Aerts and Heil [75] reported that the C/N ratio is a good predictor of the litter decomposition, particularly if the lignin content is low. In addition, the higher content of polyphenols in the pine leaves may impair the microbial activity and slows the decomposition rate [76, 77].

The dynamics of woody debris are opposite to leaf litter, in fact, they accumulate over time. In the oak plantation, woody debris represents 14% of the total litter fall, while in the litter accumulated they account for 29% because of their lower decomposition rate. A similar behavior was detected in the pine plantation, where woody debris represented 25% of total litter fall and 30% of the accumulated litter. Thus, the decomposition indexes obtained for woody materials indicated their slow decomposition rate [56].

The fine leaf litter represents an important source of organic matter and C to soil, which favors the soil biological activity and the reestablishment of nutrient cycling in degraded soils. However, while litter decomposes the organic matter and C inputs must be considered as potential returns.

Restrepo et al. [9] evaluated the potential use of both active and passive models to restore soil biogeochemical nutrient cycles through fine litterfall and soil quality in tropical degraded dry lands by overgrazing (Tables 1 and 2). They found that in degraded soils the passive restoration model with a six-year-old native species (*Croton leptostachyus*) showed a higher capacity to reestablish nutrient cycling than with an active restoration model using a six-year-old plantation of neem (*Azadirachta indica*). In these degraded soils the supply of litter by the established or successional vegetation is a valuable source of organic matter and C. In the passive model the potential C return by leaf litter represented 114 kg C ha^−1^ yr^−1^, the *k*_*j*_ constant obtained in the study was 0.6, the MRT was 1.7 yr, and the real carbon return was 72 kg ha^−1^ yr^−1^. Meanwhile, the potential C return in the active model (neem plantation) was 46 kg ha^−1^ yr^−1^ and the real C return of 33 kg ha^−1^ yr^−1^.

On the other hand, León [78] reported that in plantations of *P. patula* established in degraded highland by overgrazing the potential return of organic matter was 4866 kg ha^−1^ yr^−1^; given a *k*_*j*_ of 0.4 the real return of organic matter was 1946 kg ha^−1^ yr^−1^ (863 kg C ha^−1^ yr^−1^).

The *k*_*j*_ values in Table 2 corresponding to restoration projects are lower than those obtained from natural forests and forestry plantations, except those reported by Santa Regina and Tarazona [79] with leaf litter of forestry species in Northeastern Spain (*k*_*j*_ for *P. sylvestris = *0.31 and for *Fagus sylvatica = *0.29). Similarly, Santa Regina [80] reported *k*_*j*_ of 0.52 for litter of *Q. rotundifolia* and values of 0.26 for litter of *P. pinea* and *P. pinaster* in Duero, Spain.

## 7. Organic Matter Return Based on the Litter-Bag Approach

An alternative approach to determine the decomposition of organic matter from litter materials is through the use of the litter bag technique [22] and regression models [26]. These models describe the weight loss of a leaf litter sample (e.g., 10 g dry basis) disposed in litter-bags over time. The most common model used is the single exponential proposed by Olson [81]:

(9) $$\begin{array}{c} \frac{X_{t}}{X_{0}}=e^{-kt}, \end{array}$$

where *X*_*t*_ is the weight of the remaining material at moment *t*, *X*_0_ is the weight of the initial dry material, *e* is the base of natural logarithm, and *k* is the decomposition rate.

The amounts of time required for loss of 50% and 99% of the initial material can be calculated as *t*_50_ = −0.693/*k* and *t*_99_ = −4.605/*k*, respectively [81, 82].

**Figure 1:**
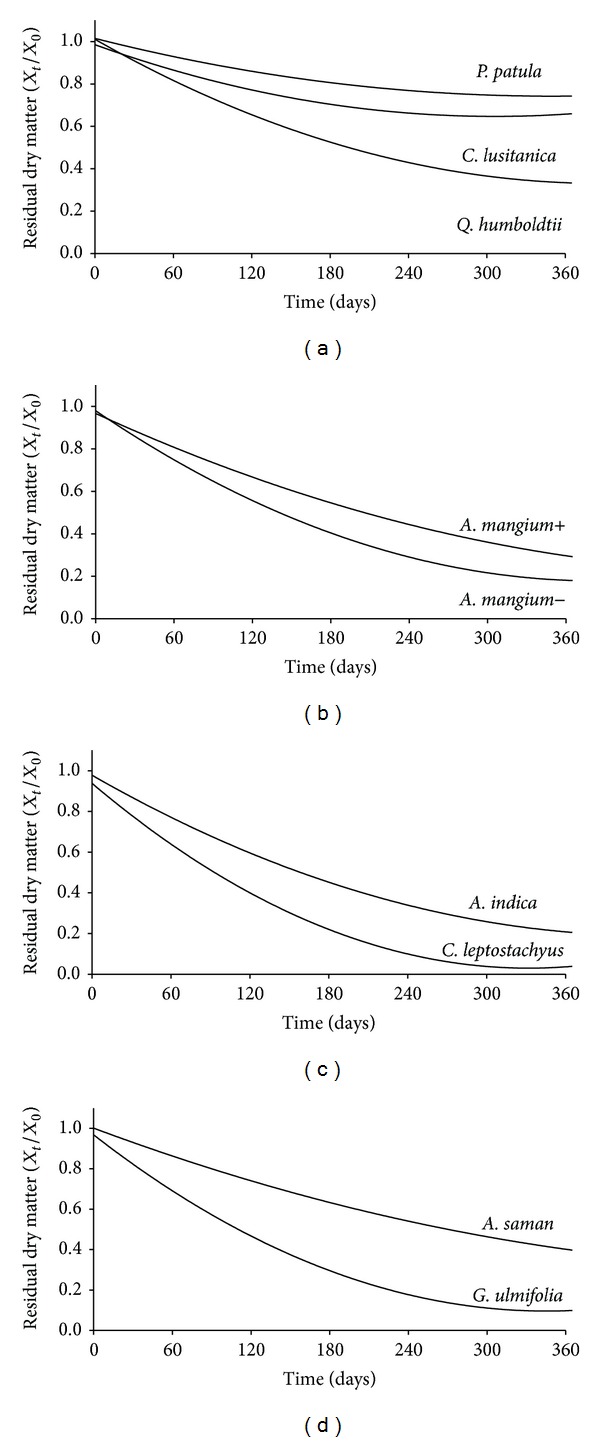
Residual dry matter (*X*_*t*_/*X*_0_) of leaf litter from restoration projects conducted in different regions of Colombia: (a) Forest of *Q. humboldtii* and plantations of *P. patula* and *C. lusitanica* in humid highlands degraded by overgrazing; (b) plantations of *A. mangium* established in degraded soils by alluvial mining (+: with soil tillage, −: without soil tillage); (c) plantation of *A. indica* and successional forest of *C. leptostachyus* in degraded dry lowlands; (d) silvopastoral systems with *A. saman* and *G. ulmifolia* in dry lowlands.

Decomposition rates obtained from restoration of different ecosystems in Colombia using this model are shown in Table 3 and Figure 1. These values correspond to values observed in tropical forests and plantations (*k* = 0.1–4.8) as those reported by several authors [67, 83, 84]. This means that the decomposition rates of litter in restoration projects are quite similar to those of plantations and forest and presumably contribute significantly in the supply of organic matter, and release of carbon and nutrients into degraded soils [10, 11].

In another project aiming to restore degraded lands by alluvial mining in Colombia, an 11-year-old plantation of *A. mangium* had annual litter fall of 10.3 Mg ha^−1^ [11, 85], 55% (5.7 Mg ha^−1^) corresponding to the leaf fraction. In this plantation the *k* from the Olson model fluctuated between 1.25 and 1.80. Therefore, the annual return of C from litter decomposition was in the range of 2.0–2.4 Mg of C ha^−1^. It is logical to consider that these values may be higher because of the restrictions imposed on the entrance of mesorganisms by the pore size of the litter-bag (~2 mm of diameter). Also, the only fraction considered in this type of study was the leaf litter.

In dry lands of Colombia, the *k* value obtained from the leaf litter of *C. leptostachyus* (*k* = 3.4) was higher than that found in the *A. indica* plantation (*k* = 1.6) [9, 58]. So, the first type of leaf litter was decomposed faster than *A. indica* leaf litter. With leaf litterfall values of 478 kg ha^−1^ yr^−1^ for the *C. leptostachyus* successional forest and 185 kg ha^−1^ yr^−1^ for the *A. indica* plantation, the potential return of organic matter into soil was 461 and 147 kg ha^−1^ yr^−1^, respectively. The value of *k* found in the *A. indica* plantation corresponded to those reported for the same plant species in restoration projects of degraded soil by mining in India [83]. In this way, the time estimated to decompose 50% and 99% of the leaf litter would be *t*_0.5_ = 0.4 yr and *t*_0.99_ = 2.91 yr, respectively.

In silvopastoral systems established in dry lowlands of Colombia Martinez et al. [24, 25] found that the outstanding plant species were *A. saman* and *G. ulmifolia* with leaf litterfall of 478 and 489 kg ha^−1^ yr^−1^, respectively. Based on their *k* values (Table 3) and C contents (45.6 and 48.8%, resp.), the potential returns of organic matter were 770 (351 kg C ha^−1^ yr^−1^) and 448 kg ha^−1^ yr^−1^ (219 kg C ha^−1^ yr^−1^), respectively.

## 8. Nutrient Return to Degraded Soil

As mentioned above, in tropical environments fine litterfall represents the main process that determines the potential return of organic matter and nutrients to the soil, which supports plant development and soil biota [86, 87]. However, nutrient recycling is achieved when the litter is decomposed by soil biota, a key process in forestry systems that determines soil quality and forest primary productivity [27]. If the nutrients are quickly released, they could be lost by leaching or volatilization [88, 89]. If the decomposition occurs slowly the nutrient supply to plant roots will be insufficient, thus limiting plant growth and development [90, 91]. For these reasons, the rates at which litter decomposition and subsequent nutrient release occur constitute key factors for ecosystem functioning. In the case of reclamation of degraded lands, this may be achieved by establishing forestry species of rapid growth, which must be selected according to their ability to adapt to extreme and restrictive soil and weather conditions. Through litter decomposition and consequent nutrient release, the vegetation can contribute to the improvement of soil quality due to their capacity to induce ecological and physicochemical changes in the soil [38, 39].

The potential nutrient return via leaf litter production in forest ecosystems has been widely reported in many studies. On the other hand, this kind of reports in restoration projects of tropical degraded lands is scarce. High values of N return via leaf litter (52–81 kg N ha^−1^ yr^−1^) were reported by León et al. [11] in *A. mangium* established in degraded soils by alluvial gold mining (Table 4). Likely, this high return is due to the ability of this plant to form symbiotic association with N_2_ fixing bacteria, which allow having high N concentration in the leaf litter (1.2%) [85]. This is a remarkable aspect desirable for a forest species selected for a soil reclamation project, as this symbiosis offers independence of the plant from the soil N reservoir.

On the other hand, the values found for the potential return of P are consistently very low in most restoration projects (0.06–1.70 kg ha^−1^ yr^−1^, Table 4). This low level of P in the leaf litter exerts a severe restriction for microbial activity and plant growth in these soils. As a consequence, low leaf litter concentrations of P can be found (0.001–0.04%), representing a major limiting factor in nutrient cycling and plant nutrition in tropical environments. The low P concentration in the litter may be a limiting factor for decomposers given their high P requirements [92]. In fact, the N/P ratio of 124 obtained in *A. mangium* litter is considerably higher than the critical value of 12 reported for the leaf litterfall [93]. For this reason, it has been proposed that inoculation with mycorrhizal fungi and phosphate solubilizing microorganisms in restoration projects of degraded land in the tropics is highly recommended [47, 94].

Similarly, in the restoration projects of degraded dry lands ecosystems with *C. leptostachyus* forests and *A. indica* plantations there was an extremely low availability of P in the soil, which was reflected in the low values of P return through leaf litter (Table 4) and in the high levels of N/P ratio (*A. indica *leaf litter: 43; *C. leptostachyus *leaf litter: 20) [9]. By the same way, in coniferous forest plantations established in highlands of Colombia, very low values for potential P return were found (0.8–1.7 kg ha^−1^ yr^−1^) [14]. Likely, it was also the result of extremely low soil P availability in the volcanic-ash soils where the study was carried out [26]. Mean values of the N/P ratio of the leaf litter of this study were 19.8 and 19.5 for pine and cypress litters, respectively. Again, these values of N : P ratio suggest a P deficiency in both materials, consequently soil reclamation could be constrained by this P deficiency.

In general, nutrient return via leaf litter follows the decreasing sequence: N > Ca > Mg > K > P; nevertheless, in some cases the potential return of Ca can be higher than that of N (Table 4). In fact, the mean concentrations of Ca and Mg in the leaf litter of *C. leptostachyus* (1.8 and 0.6%, resp.) and *A. indica* (2.2 and 0.5%) are very high and differ from those found in other tropical dry lowland forests [95]. This is the result of the high availability of both nutrients in that soil, which favored their uptake by both plant species. In contrast, the litter K concentrations found in the Neem leaf litter (ca. 0.29%) by Flórez-Flórez et al. [58] are close to the lower end of the pantropic interval (0.27 ± 0.11) suggested by Duivenvoorden and Lips [96]. This potential K scarcity may be due to the high levels of soil exchangeable Mg, which could affect the primary productivity in those plantations and, consequently, limiting leaf litter K concentrations [9]. These aspects should be carefully monitored in land reclamation projects, as they can impair the success of restoration projects.

## 9. Soil Quality Improvement in Restoration Projects

The organic matter and nutrient supply exerted by the litter production and decomposition have the potential to enhance soil properties [22]. In different projects abovementioned aiming reclamation of degraded lands we have detected increase in some soil properties such as soil organic matter (SOM), soil aggregate stability (SAS), soil total N (SN_*t*_), soil available P (SAP), and cation exchangeable capacity (CEC) (Table 5). Restrepo et al. [9] also reported a significantly decrease in the soil bulk density from 1.35 Mg m^−3^ in the control sites to 1.25 Mg m^−3^ in the planted sites with either *A. indica* or *C. leptostachyus* (both 6-year-old). This represents an increase in soil porosity and water retention, which is a major change in this dry environment. In addition, Martinez et al. [24, 25] indicated that soil exchangeable K increased from 0.78 cmol_c_ kg^−1^ in the degraded grassland up to 0.95 and 1.19 cmol_c_ kg^−1^ in the soil influenced by the litterfall of the trees *A. saman* and *G. ulmifolia *(both 13-year-old). A similar situation was detected for Ca (10.7 cmol_c_ kg^−1^ in the grassland soil and 12.4 and 13.8 cmol_c_ kg^−1^ in the soil with *A. saman* and *G. ulmifolia* litterfall, resp.).

These changes detected in soil properties represent ultimately the benefits associated with the reactivation of biogeochemical cycle via litterfall production and decomposition, which in turn will likely improve plant performance. Surprisingly, these changes were detected in relative short periods of time (6–13 years) after the establishment of the respective plantations or successional forest. Noteworthy is the significant increase in SOM reported by León et al. [10] and Restrepo et al. [9] since the SOM is key factor in soil and ecosystem functioning for plant nutrition, soil sustainability, and protection as reported by Fernandes et al. [98] in Brazil and Mafongoya et al. [99] in Africa. Likely, these changes are associated with an improvement in soil microbial activity and diversity [100].

The increases in soil available P and cation exchange capacity as a result of litter influence are outstanding, because these two properties severely impair soil quality and plant performance in the tropics. The low soil P availability seems to be one of the most critical issues for land reclamation in the tropics; in order to manage this problem the coinoculation with mycorrhizal fungi and phosphate solubilizing P may help to reduce this limitation [101]. On the other hand, soil N limitation may be offset with the combined employ of organic amendments and the massive employ of legume trees capable of forming symbiotic association with effective N_2_ fixing bacteria.

## 10. Future Research Guidelines

Currently, the strong alterations on ecosystem functions produced by soil mismanagement after the conversion of natural ecosystems into agricultural systems or mining activities are broadly accepted. These alterations have affected essential ecological processes and life support systems by breaking out biogeochemical cycles and other biosphere processes (e.g., nutrient cycling, water supply, and water regulation). Environmental and land use planning agencies in tropical countries as Colombia, where high deforestation and land degradation rates have occurred, need to develop proper knowledge of both structural and functional ecosystem parameters for measuring degrees of land degradation. Furthermore, these parameters would allow determining the effectiveness of reclamation activities on degraded lands, which can control the degradation processes. Nutrient cycling, as a major ecosystem function, provides meaningful services that have both direct and indirect benefits to future reclamation activities through active and passive strategies. Regardless the strategy employed, litter turnover and nutrient release should be carefully studied to reveal the effectiveness of the measures taken. In fact, it has been found that restoration strategies carried out in degraded lands have enhanced soil quality parameters such as increasing organic matter content and nutrient availability, regulating soil pH, improving soil aggregate stability, and providing higher water holding capacity. However, little has been done on the impact of these strategies on soil microorganisms such as mycorrhizal fungi, N_2_ fixers, mineral solubilizers, and plant growth promoters, which can enhance plant performance and soil remediation.

It has to be pointed out that these studies will demand economical resources, not always available, to make the extended monitoring of processes abovementioned viable. The lack of these financial resources should be overcome by state agencies through their science and technology systems as well as some private institutions by means of fiscal mechanisms to encourage their participation in developing research and applied programs in degraded land reclamation.

## 11. Conclusions

The organic matter and nutrient return rate via litterfall depends on many factors that influence decomposition process at the ecosystem level. Plant species selected for reclamation of degraded soils should preferably be able to establish symbiotic associations with soil microorganisms (i.e., mycorrhizal fungi and N_2_ fixing bacteria). Furthermore, these species must show a high capacity to adapt to nutrient-poor acidic soils. Because microbial activity is severely restricted in degraded soils, the selected species must have high nutrient use efficiency. The reclamation of degraded soil requires deep knowledge of the litter dynamics (e.g., litter fall, nutrient content, and decomposition rate) because this determines the rate of organic matter and C supply to the soil and the nutrient cycling reactivation. In relatively short periods of time it is possible to detect the improvement of some soil properties due to litter fall and decomposition. Despite that, low soil availability of N and P seems to be the major constraints in reclamation of degraded soil in the tropics, for which the use of legume trees and inoculation with beneficial microorganisms (e.g., N_2_ fixing bacteria, mycorrhizal fungi, and mineral solubilizing microorganisms) should be an integral part of the management of these fragile ecosystems.

## Figures and Tables

**Table 1:** General information on the experimental sites in Colombia.

Ecological life zone^a^	Geographic coordinates	Temperature (°C)	Precipitation (mm yr^−1^)	Altitude (m)	Land uses	Source
Site: Piedras Blancas. Degradation type: overgrazing-deforested. Soil: Typic Hapludand.^b^						
LMMF	06°18′N 75°30′W	14.9	1948	2460	*P. patula* plantation	[26, 52]
				2440	*C. lusitanica *plantation	
				2480	*Q. humboldtii *forest	

Site: Santa Fe de Antioquia. Degradation type: overgrazing-severely eroded. Soil: Typic Ustorthents.						
TDF	06°54′N 75°81′W	26.6	1034	560	*C. leptostachyus * succession forest	[9, 58]
					*A. indica* plantation	

Site: Cáceres. Degradation type: alluvial mining. Soil: Typic Paleudult.						
TWF	07°45′N 75°14′W	28.0	2771	330	*A. mangium *plantation	[10, 11]

Site: Cereté. Degradation type: overgrazing-compacted soil. Soil: Fluvaquentic Endoaquepts.						
TDF	8°51′N 75°49′W	28.0	1380	18	*A. saman and G. ulmifolia *in silvopastoral systems	[24, 25]
					Degraded grassland with *D. aristatum *and* P. maximum *	

^a^Holdridge [59] (LMWF: lower montane moist forest, TDF: tropical dry forest, TWF: tropical wet forest).

^b^USDA soil taxonomy [60].

**Table 2:** Indices calculated for leaf litter potential return (*A*), leaf litter accumulation (*F*), leaf litter real return (LLRR = *Ak*_*j*_), and real return of C (RRC = LLRR ×  C content ((%)) (expressed in Mg ha^−1^ yr^−1^) in restoration projects conducted in Colombia. MRT_*j*_: mean residence time (yr) and *k*_*j*_: decomposition constant (yr^−1^). *F* values do not include the contribution of roots.

Ecosystem	*A*	*F*	*k*_*j*_	MRT_*j*_	LLRR	RRC	Source
	(Mg ha^−1^ yr^−1^)		(yr^−1^)	(yr)	(Mg ha^−1^ yr^−1^)		
Forest of *Q. humboldtii *	5.313	3.828	0.58	1.72	3.088	1.257	[52, 78]
*P. patula* plantation	4.866	6.597	0.42	2.36	2.066	0.916	[52, 78]
Succession of *C. leptostachyus *	0.478	0.239	0.67	1.50	0.320	0.079	[9]
*A. indica* plantation	0.185	0.073	0.72	1.39	0.130	0.034	[9, 58]

**Table 3:** Fitted models for residual dry matter (*X*_*t*_/*X*_0_) as a function of time for forest species from restoration projects in Colombia.

Plant species	*k*	*t*_0.5_ (yr)	*t*_0.99_ (yr)	*R* ^2^	Source
*Q. humboldtii *	1.02	0.68	4.51	93.3	
*P. patula *	0.29	2.37	15.77	88.5	[26]
*C. lusitanica *	0.37	1.90	12.62	53.1	
*C. leptostachyus *	3.36	0.21	1.37	96.76	[9]
*A. indica *	1.58	0.44	2.91	84.31	[9]
*A. mangium *	1.35–1.80	0.38–0.56	2.60–3.70	86.82–96.20	[11]
*A. saman *	0.96	0.72	4.78	92.3	[24, 25]
*G. ulmifolia *	2.47	0.28	1.86	89.0	

*t*_0.5_: decomposition time for half of the leaf litter, *t*_0.99_: decomposition time for 99% of the leaf litter, *k*: yearly decomposition rate, *R* ^2^: coefficient of determination (%).

**Table 4:** Potential return of nutrients via leaf litterfall from the dominant plant species in different ecosystems obtained from restoration projects conducted in Colombia.

Ecosystem/ecological life zone	Nutrient return					Source
	N	P	Ca	Mg	K	
	(kg ha^−1^ yr^−1^)					
*P. patula* plantation	44.4	1.7	18.8	4.5	3.6	[52]
*C. lusitanica *plantation	13.2	0.8	26.1	1.4	1.7	[52]
Succession of *C. leptostachyus *	5.2	0.22	8.4	2.8	1.3	[9, 58]
*A. indica *plantation	2.4	0.06	4.6	0.9	0.5	[9, 58]
*A. mangium *plantation	52–81	0.3–0.8	24–35	6–9	7–13	[10, 11]
*A. saman* in silvopastoral system	34.8	1.0	12.2	1.5	4.3	[24, 25]
*G. ulmifolia* in silvopastoral system	11.2	0.7	16.1	2.1	3.8	[24, 25]

**Table 5:** Changes in some soil properties of degraded soils in Colombia by the establishment of forestry species.

Site conditions and reclamation strategy	Soil pH	SOM (%)	SAS (%)	SNt (%)	SAP (mg kg^−1^)	CEC (cmol_c_ kg^−1^)	Source
Degraded land by alluvial mining							
Unplanted control	5.4	6.1	73.0	0.24	2.5	6.1	[10, 11]
11-year-old plantation of *A. mangium *	4.5*	18.7*	85.4*	0.50*	6.5*	11.2*	

Degraded land by overgrazing							
Unplanted control	6.3	2.0	73.0	0.21	3.3	13.0	[9, 58]
6-year-old plantation of *A. indica *	6.4	3.4*	80.1	0.27*	4.3*	14.8	
6-year-old forest of *C. leptostachyus *	6.3	4.2*	68.5	0.25	1.8	25.9*	

Degraded land by overgrazing							
Degraded grassland	5.5	8.9	ND	ND	9.6	21.7	[24, 25]
13-year-old* A. saman* in silvopastoral system	5.8*	8.4	ND	ND	14.0*	22.3	
13-year-old* G. ulmifolia* in silvopastoral system	6.2*	9.0	ND	ND	24.1*	24.5*	

Analytical methods are available in Westerman [97]: soil pH (water, 1 : 1), SOM (Walkley and Black): soil organic matter, CEC (1 M ammonium acetate): cation exchange capacity, SAS (Yoder method): soil aggregate stability, SAP (Bray II): soil available P. *Significant difference with control sites (Mann-Whitney, *P* ≤ 0.05). ND: not determined.
